# Nice Finish to 2014 and Looking Forward to 2015

**DOI:** 10.5772/60139

**Published:** 2015-01-01

**Authors:** Markus Dettenhofer, Winston Patrick Kuo

**Affiliations:** 1 CEITEC Executive Director, Brno, Czech Republic; 2 IES Diagnostics, Inc, Cambridge, USA

**Keywords:** Nanotechnology, biomedical research, diagnostics, drug development, delivery, vaccines, regenerative medicine, editorial

## Abstract

This editorial article summarizes last year's achievements and current plans for 2015 that focuses on attracting a high quality, variety of articles with more emphasis in engaging with academia and industry in the field of nanotechnology and biomedical research.

During the past year, we are proud to announce several developments, first is our world class Associate Editors and Editorial Board members that are key opinion leaders in their respective expertise in the field of nanobiomedicine. We are currently continuing to expand our Editorial Board, however we would like to expand the expertise in the diagnostics, vaccine development/implementation and regenerative medicine space, as the fields are very broad-based.

NBM Editor-in-Chief and editorial board members were active in several events that took place in Boston and in Czech Republic. The two events in Boston included the “Micro- and Nanotechnologies for Medicine: Emerging Frontiers and Applications” (BioMEMS) workshop hosted by the journal's Editorial Board Member, Dr. Ali Khademhosseini and the other was “Science Shaping Our World”, held at the Merck Research Laboratories, where two of our NBM Editorial Board Members, Omar A. Ali and Qimin Quan presented. NBM's Editor-in-Chief, Dr. Markus Dettenhofer – CEITEC Executive Director, took an active part in organizing the annual “Conference Brno 2014: Frontiers in Material and Life Sciences”, sponsored by his institution, CEITEC (Central European Institute for Technology).

Lastly, Barbara S. Smith, Ph.D. (Arizona State University, USA), one of our Editorial Board members, is currently the Guest Editor of an ongoing Special Issue which was launched in October 2014, entitled “Nanobiomedicine – Meeting the Needs of Health Care across the Globe”. This special issue is devoted to publishing the latest significant innovative results in the global health spectrum – through solutions tuned for different regions throughout the world; encouraging research that uses new methods of disease diagnosis, including technological and product development for enhanced point-of-care and personalized health care. The aim of this edition is to spur both excitement and knowledge, in and out of the field, helping to open new market opportunities towards shifting cost and use paradigms. Submissions of scientific results that highlight the current path – from the research bench to the hands of the user – by technical experts within both academia and industry are welcome. (http://www.intechopen.com/journals/invitation/snbm-nanobiomedicine-meeting-the-needs-of-health-care-across-the-globe)

We are quite pleased with the number of high quality publications since the launch of NBM. The majority of papers discuss research around nanoparticles and their applications. We will reach out to other areas including, but not limited to the following areas; bioengineering, biophysics, biochemistry, physiology, pathology, nano-technological applications in diagnostics, therapeutic application, vaccine, drug discovery/delivery and regenerative medicine.

NBM accepts original and review articles as well as editorials, perspectives, short research reports, protocols and methods, letters to the editor and meeting dispatch reports.

We would like to mention, Intech publishers have decided that NBM will not apply any article processing charges for authors whose research papers have been accepted for publication in Volume 2/2015. The NBM manuscript processing and peer-review is conducted entirely online. The Editorial Manager facilitates the manuscript processing time, reducing costs and is a better experience for our authors and reviewers. We have received numerous positive comments on this evolving electronic system, and we are indebted to Intech publishers for managing this program for us. For more information, please visit our new manuscript submission system at http://www.editorialmanager.com/nmnt/. In addition, we are enforcing to expedite the acceptance to publishing time for our authors and will request informal opinions from our Editorial Board for borderline cases.

In addition, we are pleased to have established a partnership/collaboration with the BioPharma Research Council (http://www.biopharmaresearchcouncil.org/), spearheaded by Joanne Gere, the Executive Director. The BioPharma Research Council has been developing a vibrant community for scientists as they navigate today's complex environments. Our unique resources – including deep relationships with leading companies and suppliers – and agile approaches have established continual opportunities for breakthrough discussion and debate throughout the drug discovery and delivery pipeline. We plan to launch 2 webinar series, one on point of care diagnostics and the other in drug delivery later this year and are currently looking for presenters. A meeting dispatch will be published in NBM after each series.

NBM will continue to work on partnering with other scientific societies, academic and industry leaders to develop and advance the nanotechnology and biomedical research field and to allow free access to the readers.

We finally would like to thank InTech publisher for supporting the initiatives of this journal and acknowledge Petra Nenadic and her team for her continuous support, passion and dedication for making this possible. In addition, we would like to thank our highly renowned and respected Associate Editors and Editorial Board members from all over the world, whose expertise range from nano-fluidic systems to nano-scale drug delivery to nano-imaging that are bridge and a catalyst in their respective fields of nanotechnology and biomedical research.

